# Sociodemographic and clinical predictors for COVID-19 preventive measures compliance among pregnant women in Saudi Arabia: a multicenter study

**DOI:** 10.1186/s12879-023-08364-z

**Published:** 2023-06-13

**Authors:** Ranya A. Ghamri, Kholoud A. Ghamri

**Affiliations:** 1 Family Medicine Department, Faculty of Medicine, King Abdulaziz University, Jeddah, Saudi Arabia; 2 Internal Medicine Department, Faculty of Medicine, King Abdulaziz University, Jeddah, Saudi Arabia

**Keywords:** Pregnant woman, COVID-19, Compliance, Risk prevention, Fetal, Social determinants, Maternal

## Abstract

**Objective:**

To assess the levels of adherence among pregnant women to the basic COVID-19 preventive measures, and to analyze the effect of risk perception and sociodemographic and clinical factors on adherence.

**Method:**

A multicenter, cross-sectional study was conducted at the obstetrics clinics of 50 primary care centers selected using a multistage sampling method. An online-administered, structured questionnaire was used to collect self-reported levels of adherence to four basic preventive measures against COVID-19, along with perceived COVID-19 severity, infectiousness, and harmfulness to the baby, besides sociodemographic and clinical data including obstetrical and other medical history.

**Results:**

A total of 2460 pregnant women were included with a mean (SD) age of 30.21 (6.11) years. Levels of self-reported compliance were highest for hand hygiene (95.7%), followed by social distancing (92.3%), masking (90.0%), and avoidance of contact with a COVID-19 infected person (70.3%). Perceived COVID-19 severity and infectiousness, and harmfulness to the baby were observed in 89.2%, 70.7%, and 85.0% of the participants, respectively, and were variably associated with compliance to preventive measures. Analysis of sociodemographic factors highlighted the significance of education and economic status in determining adherence to preventive measures, which represents a potential inequity in the risk of COVID-19 infection.

**Conclusion:**

This study highlights the importance of patients’ education to enable functional perception of COVID-19 that promotes self-efficacy, besides investigating the specific social determinants of health to tackle inequalities in terms of prevention efficiency and the subsequent health outcomes.

## Introduction

The Coronavirus disease 2019 (COVID-19), is the most spectacular pandemic in the 21st century, changing the lives of the 7.96 billion world’s population in an unprecedented manner. It is caused by a highly transmissible subtype of coronavirus, the severe acute respiratory syndrome coronavirus 2 (SARS-CoV-2), with high cardiovascular tropism causing significant cardiovascular morbidity, besides respiratory syndrome [1, 2]. Two years after the first discovery of the virus in Wuhan, China [3], the number of COVID-19 cases is estimated to be more than 554 million individuals, with 6.3 million deaths worldwide [4]. Additionally, the social and economic impact of the pandemic is beyond any estimates, causing deep disruption to the health systems, global trade, and psychosocial wellbeing of individuals with a long-term expected knock-on effect [5–7].

The era of COVID-19 was also marked by the globally implemented preventive measures such as social distance, quarantine of infected individuals, universal masking and hand hygiene. These measures were highly effective in controlling the spread of virus and flattening the epidemic curve [8, 9]. These were combined with a plethora of public health interventions, such as awareness messages and educational programs, to enhance people’s adherence to the preventive measures, while mitigating their anxiety and fear [10, 11]. These measures were continued at variable levels and durations even after the implementation of vaccination, to maximize the effectiveness of the preventive strategy.

Substantial researches have been conducted to study the adherence to preventive measures in different populations to elucidate the socio-demographic, cognitive, and behavioral determinants of non-adherence to inform the decision makers on how to optimize the preventive strategies [12, 13]. Pregnant women are one of the concerned specific populations. The pathophysiological mechanisms of COVID-19 suggest a high risk for both mother and fetus, notably due to the increased expression of angiotensin converting enzyme 2 (ACE2) receptors during pregnancy, while these receptors represent the entry gate for SARS-CoV-2 in the human cells [14, 15]. On the other hand, clinical evidence showed mixed results regarding the increased maternal or fetal risk in case of COVID-19 infection during pregnancy. Several reports suggested increased risk of pre-eclampsia, prematurity, and perinatal death. Such complications were observed in women infected at an early gestational age, as well as those who did not receive adequate ventilator support during the acute phase [16–18]. However, a systematic review that included approximately 11,000 cases from 15 different countries concluded the absence of increased risk among mothers or fetuses due to COVID-19 in comparison to the general population [19]. Nevertheless, pregnant women remain particularly vulnerable to severe forms of COVID-19, which results in high mortality and longer hospitalization [20, 21]. This emphasizes the importance of improving primary prevention to avoid the excess comorbidity among this subpopulation. On the other hand, several sociodemographic and health-related factors may interfere with the pregnant women’s attitudes towards and adherence to COVID-19 preventive measures.

We conducted this study to assess the levels of adherence among pregnant women to the basic COVID-19 preventive measures, and to explore the association of adherence with COVID-19 risk perception. We also analyzed the socioeconomic and obstetrical determinants of adherence to preventive measures in order to determine the significant predictors and inequalities that would inform on the optimal preventive strategy to implement among this vulnerable group.

## Methods

### Design and participants

This was a multicenter, clinic-based cross-sectional study carried out during July – September 2021. It targeted all national and resident pregnant women who visited the primary care obstetrical clinics during the study period, for antenatal care and follow up of their pregnancy. Antenatal care represents one of the priority health services of the Saudi Government. It starts in the first trimester and is provided throughout the pregnancy, and comprises of at least eight appointments for an uncomplicated pregnancy. As the study was part of a bigger research project exploring the acceptance of COVID-19 vaccination among pregnant women [22], participants who were not eligible to receive the vaccine were excluded; this applied for women aged below 18 years and those having a contraindication to COVID-19 vaccination (i.e., allergy to vaccine components).

### Sampling

Saudi Arabia is divided into 13 Regions, each Region contains a number of Governorates (3 to 21) for a total of 136 Governorates in the Kingdom. A multistage sampling method was used. In Stage one, the 13 regions of Saudi Arabia were stratified into five geographic sections, including the Northern (Tabuk, Al Jawf, and Northern Borders [total 12 Governorates]), Southern (Asir, Jizan, and Najran [39 Governorates]), Eastern (Eastern Province [11 Governorates]), Western (Al Madinah, Makkah, and Al Baha [total 33 Governorates]), and Center (Hail, Al Qassim, and Riyadh [total 41 Governorates]). In stage two, two Governorates were selected from each geographic section, using random sampling method. This resulted in 10 Governorates selected out of a total of 136. In stage three, each selected Governorate was further subdivided into 5 sectors (North, South, East, West, and Center) and one primary care center was selected from each sector using a random sampling process. This resulted in 5 PHCs from each Governorate, 10 PHCs from each geographic section, and a total 50 PHCs from across the Kingdom.

Sample size was calculated to detect an unknown compliance rate with preventive measures (P = 0.5) among a large population (> 1,500,000), within each geographic section, with 95% confidence interval (95% CI), 0.05 margin error and 80% statistical power. The sample size for each geographic section was estimated as 385 participants, for a total N = 1925 participants. The target sample size was increased by 25% to compensate eventual incomplete participations or drop-outs, thus increased to N = 2410, that is 482 participants from each geographic section. All eligible and consenting pregnant women who visited the antenatal care clinic in the participating PHCs were invited to participate until reaching the total sample size.

### Tools

A brief, structured questionnaire was designed by the authors and edited in both English and Arabic languages. It comprised of four compartments:

1. Socio-demographic data including patient’s age, region of residency, nationality, educational level, career (housewife vs. employed in private or governmental sector), husband’s career, economic status based on household income (low, average, and high), number of householders, number of school-aged children and seniors (65 years-old and above) in household.
2. Obstetrical and other clinical characteristics including gravidity, parity, pregnancy trimester based on gestational week, high-risk status (no/yes) of the pregnancy based on gestational conditions (gestational diabetes, hypertension, multifetal pregnancy, etc.), and any other medical comorbidity (diabetes, heart disease, etc.), in addition to vaccinations for tetanus and influenza during the current pregnancy, and any other vaccine in the past 5 years.
3. Self-assessed compliance with major preventive measures against COVID-19, including avoidance of contact with an infected or suspected person, hand hygiene, social distancing, and masking. Each of the dimensions was assessed using a dichotomous answering option, “yes” for adequate compliance and “no” for inadequate compliance.
4. Risk perception about COVID-19 comprising of three statements about perceived severity “I believe COVID-19 is a serious disease”, perceived infectiousness “I believe I have lower risk of COVID-19 infection”, and perceived vulnerability for the baby “I believe that, even if I am sick, COVID-19 will not have negative effects on my baby”. There were two answering options for each statement, “true” or “false”.

The questionnaire underwent face and content validity, with the contribution of an epidemiologist in addition to two physicians and two nurses with expertise in antenatal care and significant experience in clinical research. The revised and improved version of the questionnaire underwent pilot testing, which was carried out among 10 pregnant women visiting pregnancy follow up clinics, to assess the clarity and convenience of the questionnaire length. The final version was edited and adapted in both English and Arabic languages by bilingual authors.

### Data collection procedures

The questionnaire was edited for an online administration on June 2021. The authors assigned data collectors in each region. The data collectors were trained to reach the target population at the participating clinics; they approached the attending pregnant women; assessed their eligibility for participation, explained the study objectives, collected verbal consent for participation, and finally provided the questionnaire link by phone text message to eligible and consenting women. The filled questionnaires were automatically collected via the online software platform, and the final database was extracted as an Excel sheet. The questionnaire link was kept active during the study period and until the target sample size was reached; afterwards, the link was inactivated.

### Ethical clearance

The questionnaire was designed anonymously, without collecting identifying data. All participants provided their verbal consent prior the interview. Data was collected, processed and stored with confidentiality. Ethical approval was obtained from the ethical committee of King Abdulaziz University (Reference No 439 − 22), and research was performed according to the principles of Declaration of Helsinki for medical research. All respondents provided digital informed consent before the questionnaire was administered.

### Statistical methods

Data was collected, coded and cleaned using Microsoft Excel. The cleaned database was transferred to the Statistical Package for Social Sciences version 21.0 for Windows (SPSS Inc., Chicago, IL, USA) for data analysis. Descriptive statistics were carried; categorical variables are presented as frequency and percentage, while continuous variables are presented as mean ± standard deviation (SD).

Reliability analysis of the 4 compliance items showed a low Cronbach’s alpha of 0.450. Therefore, each preventive measure was analyzed separately. A compliance rate was calculated for each preventive measure, as the percentage of participants who replied adequately adhering to the given measure. Chi-squared test was used to analyze the association of compliance with the different sociodemographic, obstetrical and other clinical factors, as well as with the risk perception levels; results are presented as the compliance rate for each preventive measure, within each category of the factor. For risk perception levels, a compliance ratio was calculated as the compliance rate among participants who replied “true” to the risk perception statement divided by the compliance rate among those who replied “false”. A compliance ratio > 1 indicates that belief that the given statement is true is associated with a better compliance to the given preventive measure.

To analyze the independent factors of compliance, a multivariate logistic regression (Enter method) model was carried out for each preventive measure (the dependent variable), by including all factors that showed statistical significance in the chi-squared analysis. Dummy variables were used for specific categories that showed significant differences with the other categories of the same multinomial factor. Results of the regression models are depicted as odds-ratio (OR) with 95% CI and the level of statistical significance for each factor of dummy variable.

A p-value of < 0.05 was considered to reject the null hypothesis.

## Results

### Participants’ characteristics

Two thousand four hundred and sixty questionnaires were adequately completed. The mean (SD) age of the participants was 30.21 (6.11) years. Distribution by region showed that the Northern and Southern regions were underrepresented accounting for 10.5% and 8.8% of the total participants, respectively. Other demographic characteristics showed a high percentage of housewives (65.9%) despite 78.9% had university level education (Table 1).

**Table 1:** Participants’ socio-demographic characteristics (N = 2460)

Parameter	Unit	Mean	SD
Age	(years)	30.21	6.11
**Parameter**	**Category**	**Frequency**	**Percentage**
Region	Western Province	714	29.0
	Eastern Province	694	28.2
	North Province	258	10.5
	South Province	216	8.8
	Central Province	567	23.0
	Not specified	11	0.4
Nationality	Saudi	2348	95.4
	Non-Saudi	112	4.6
Education	None	16	0.7
	Primary school	66	2.7
	Secondary school	436	17.7
	University	1942	78.9
Career	Housewife	1622	65.9
	Private sector	322	13.1
	Government official	516	21.0
Husband career	Not Working	138	5.6
	Private sector	820	33.3
	Government sector	1405	57.1
	Merchant	97	3.9
Household income	Less than 3000 SR	321	13.0
	3000–8000 SR	959	39.0
	More than 8000 SR	1180	48.0
No. householders	1	101	4.1
	2	646	26.3
	3–4	875	35.6
	5+	838	34.1
No. school-age children in household	0	1249	50.8
	1	423	17.2
	2+	788	32.0
No. seniors in household (65 years old and above)	0	2189	89.0
	1	175	7.1
	2+	96	3.9

SD: Standard deviation; SR: Saudi Riyal

Obstetrical characteristics showed high gravidity (69.8% having gravida ≥ 2) and high percentage of multiparity (47.7%). Regarding pregnancy stage, 51.7% of the participants were in the third trimester and 13.2% in first trimester. Morbidity showed prevalence of high-risk pregnancy (14.7%) and other medical conditions (9.2%) which included diabetes (2.8%) and hypertension (1.1%) (Table 2).

**Table 2:** Participants obstetrical and other medical characteristics (N = 2460)

Parameter	Unit	Mean	SD
Gravidity	(N)	2.83	1.97
Parity	(N)	1.79	1.73
Gestational week	(weeks)	25.81	10.13
**Parameter**	**Category**	**Frequency**	**Percentage**
Gravidity	0	20	0.8
	1	722	29.3
	2–3	1007	40.9
	4+	711	28.9
Parity	0	653	26.5
	1	633	25.7
	2–3	805	32.7
	4+	369	15.0
Trimester	First trimester (0–13 weeks)	324	13.2
	s trimester (14–26 weeks)	863	35.1
	Third trimester (27–40 weeks)	1273	51.7
High-risk pregnancy	No	2099	85.3
	Yes	361	14.7
	*Gestational diabetes mellitus*	*134*	*5.4*
	*Preterm labor*	*84*	*3.4*
	*Gestational hypertension*	*46*	*1.9*
	*Multifetal pregnancy*	*45*	*1.8*
	*Placenta previa*	*39*	*1.6*
	*Fetal structural anomalies*	*12*	*0.5*
	*Epilepsy*	*1*	*0.0*
Medical condition	No	2234	90.8
	Yes	226	9.2
	*Diabetes*	*70*	*2.8*
	*Hypertension*	*28*	*1.1*
	*Heart disease*	*10*	*0.4*
	*Other*	*118*	*4.8*
Any vaccination in the past 5 years	No	1808	73.5
	Yes	652	26.5
Influenza vaccine during current pregnancy	No	2330	94.7
	Yes	130	5.3
Tetanus vaccine during current pregnancy	No	2291	93.1
	Yes	169	6.9

### COVID-19 risk assessment and compliance with preventive measures during pregnancy

Levels of self-reported compliance with COVID-19 preventive measures was highest for hand hygiene (95.7%), followed by social distancing (92.3%), masking (90.0%), and avoidance of contact with a COVID-19 infected person (70.3%) (Table 3).

**Table 3:** COVID-19 risk assessment and compliance with preventive measures during pregnancy

Type of risk	Level	Frequency	Percentage
Avoided close contact with a COVID-19 infected person	No	731	29.7
	Yes	1729	70.3
Compliance with hand hygiene	No	106	4.3
	Yes	2354	95.7
Compliance with social distancing	No	190	7.7
	Yes	2270	92.3
Compliance with masking	No	245	10.0
	Yes	2215	90.0

### Risk perception and its association with compliance to COVID-19 preventive measures

Only 1697 (69.0%) of the participants have completed the risk perception questionnaire. Of them, 89.2% believed that COVID-19 is a serious disease, 29.3% believed having lower risk of being infected, and 15.0% believed that COVID-19 would not harm the baby’s health.

Believing that COVID-19 is a serious disease increased the compliance rate by up to 1.23-fold depending on the preventive measure, and the results were statistically significant for all 4 preventive measures (p < 0.001). Believing that COVID-19 would not harm the baby decreased the compliance rate by up to 13%, depending on the preventive measure, and was significant for avoidance of contact with a COVID-19-infected person (p = 0.003) and mask wearing (p = 0.032). No significant effect was observed for the level of perceived infectiousness on compliance rates (Table 4).

**Table 4:** Risk perception and its association with compliance to COVID-19 preventive measures (N = 1697)

Risk perception level	N (%)	COVID-19 prevention measures, compliance rate			
		Avoid contact with infected person	Hand hygiene	Social distancing	Masking
**I believe COVID-19 is a serious disease**					
False	183 (10.8)	57.9%	88.0%	84.7%	78.1%
True	1514 (89.2)	71.3%	96.3%	92.9%	90.3%
*Compliance ratio (p-value)* ^*§*^		1.23 (< 0.001*)	1.09 (< 0.001*)	1.10 (< 0.001*)	1.16 (< 0.001*)
**I believe I have lower risk of COVID-19 infection**					
False	1199 (70.7)	68.7%	94.4%	90.7%	88.2%
True	498 (29.3)	72.5%	97.8%	95.2%	91.0%
*Compliance ratio (p-value)* ^*§*^		1.23 (0.124)	1.04 (0.002*)	1.05 (0.002*)	1.03 (0.093)
**I believe that, even if I am sick, COVID-19 will not have negative effects on my baby**					
False	1442 (85.0)	71.2%	95.6%	92.2%	89.7%
True	255 (15.0)	62.0%	94.5%	91.4%	85.1%
*Compliance ratio (p-value)* ^*§*^		0.87 (0.003*)	0.99 (0.460)	0.99 (0.667)	0.95 (0.032*)

Compliance ratio is calculated as the compliance rate among those who believe the risk perception is true (nominator) divided by the compliance rate among those who believed the risk perception is false (denominator). Thus, it expresses the relative compliance for the “true” level of risk perception, which is considered “protective” for a compliance ratio > 1 and “deleterious” if compliance ratio < 1

Test used: Chi squared test; * statistically significant difference (p < 0.05)

### Sociodemographic and clinical factors associated with compliance to COVID-19 preventive measures

Several statistically significant associations were observed between compliance with the different preventive measures and sociodemographic and clinical factors; these are depicted in Table 5. For example, participants from the Northern province had lower compliance rate with avoidance of contact with a COVID-19-infected person (p < 0.01). Both lower education and lower economic status were associated with lower compliance rates with different preventive measures. Obstetrical factors showed mixed results and a few statistically significant results, with no obvious clinical impact.

**Table 5:** Socio-demographic and clinical factors associated with compliance to COVID-19 preventive measures (N = 2460)

Factor	Level	COVID-19 prevention measures, compliance rate (%)			
		Avoid contact with infected person	Hand hygiene	Social distancing	Masking
Age (years)	≤ 30	70.9	95.1	91.4	89.9
	> 30	69.4 ^NS^	96.5 ^NS^	93.4 ^NS^	90.3^NS^
Region	Western Province	74.6	96.9	93.1	92.2
	Eastern Province	67.9	95.7	92.4	90.3
	North Province	60.5**	96.9	89.9	88.8
	South Province	74.1	94.4	92.6	88.9
	Central Province	70.9	94.2	92.4	88.0
	Not specified	63.6	90.9 ^NS^	72.7 ^NS^	90.0 ^NS^
Nationality	Saudi	70.4	95.7	92.3	89.9
	Non-Saudi	68.8 ^NS^	96.4 ^NS^	91.1 ^NS^	92.0 ^NS^
Education	None	75.0	81.3**	62.5**	68.8*
	Primary	62.1*	92.4	86.4	90.9
	Secondary	76.1	94.0	93.3	90.1
	University	69.2	96.3	92.5	90.2
Career	Housewife	72.1*	95.3	92.0	89.1
	Private sector	64.9	95.0	91.3	91.3
	Government official	67.8	97.5 ^NS^	93.8 ^NS^	92.1 ^NS^
Husband career	Unemployed	71.7	91.3	85.5	84.1
	Private sector	70.5	95.4	92.3	90.0
	Government sector	70.2	96.5	93.1	91.2
	Self-employed	67.0 ^NS^	92.8*	89.7*	82.5**
Household income (SR)	Less than 3000	72.9	92.5**	88.2**	83.5**
	3000–8000	71.8	95.6	91.7	89.9
	More than 8000	68.3 ^NS^	96.6	93.9	91.9
No. householders	1	71.3	98.0	85.1*	87.1**
	2	72.1	95.4	92.6	92.4
	3–4	70.5	95.9	92.1	87.5**
	5+	68.5 ^NS^	95.5 ^NS^	93.1	91.2
No. school-age children in household	0	70.6	95.2	91.4	89.8
	1	69.3	95.7	92.2	88.4
	2+	70.3 ^NS^	96.4 ^NS^	93.7 ^NS^	91.2 ^NS^
No. seniors in household	0	71.0	95.9	92.7	90.1
	1	68.0	95.4	88.6	92.0
	2+	58.3*	90.6*	89.6 ^NS^	85.4 ^NS^
Gravidity	0	80.0	80.0**	85.0	90.0
	1	71.9	95.2	91.0	91.6
	2–3	69.1	95.3	92.9	87.7*
	4+	70.0 ^NS^	97.2	93.0 ^NS^	91.8
Parity	0	70.4	94.5	91.1	91.7
	1	70.8	94.9	91.9	88.3
	2–3	70.2	97.0	92.9	88.7
	4+	69.4 ^NS^	96.2 ^NS^	93.5 ^NS^	93.0*
Trimester	First trimester	77.5**	93.5%	91.7	91.0
	s trimester	68.1	96.1	94.1	91.9
	Third trimester	69.9	96.0 ^NS^	91.2*	88.5*
High-risk pregnancy	No	70.7	95.7	92.2	89.9
	Yes	67.9 ^NS^	95.6 ^NS^	92.8 ^NS^	90.6 ^NS^
Medical condition	No	70.9	95.7	92.6	90.0
	Yes	64.2*	95.1 ^NS^	88.9*	90.3 ^NS^

Test used: Chi squared test; NS not statistically significant; * p<0.05; ** p<0.001

### Predictors of compliance to COVID-19 preventive measures

Multivariate regression models for the predictors of compliance to preventive measures are depicted by specific measures in Table 6. Compliance to avoidance of contact with an infected person was independently associated with housewife status (OR = 1.33, p = 0.002) and being in the first trimester of pregnancy (OR = 1.52, p = 0.003) in a positive relationship, and with residence in Northern Province (OR = 0.60, p < 0.001) and having two or more seniors in household (OR = 0.57, p = 0.008) in a positive relationship. Compliance with hand hygiene was independently associated with low economic status (OR = 0.59, p = 0.047) and nulligravida (OR = 0.24, p = 0.021). Compliance to social distancing was independently associated with no formal education (OR = 0.20), living alone in household (OR = 0.48, p = 0.013), and second trimester of pregnancy (OR = 1.54, p = 0.012). Compliance with masking was independently associated with no formal education (OR = 0.32, p = 0.048), self-employed husband (OR = 0.52, p = 0.022), and low economic status (OR = 0.56, p = 0.002).

**Table 6:** Predictors of compliance to COVID-19 preventive measures (multivariate logistic regression)

Preventive measure (Dependent variable)	Predictor	OR	95% CI		p-value
Avoid contact with infected person	**Residence in Northern province**	**0.60**	**0.46**	**0.79**	< 0.001*
	Primary school level	0.73	0.44	1.22	0.233
	**Housewife**	**1.33**	**1.11**	**1.60**	0.002*
	**2 or more seniors in household**	**0.57**	**0.37**	**0.86**	0.008*
	**First trimester pregnancy**	**1.52**	**1.15**	**2.01**	0.003*
	Medical comorbidity	0.75	0.56	1.00	0.052
Hand hygiene	No formal education	0.27	0.07	1.04	0.057
	Unemployed husband	0.76	0.36	1.62	0.477
	Self-employed husband	0.62	0.27	1.40	0.245
	**Low income (< 3 K SAR)**	**0.59**	**0.35**	**0.99**	0.047*
	2 or more seniors in household	0.50	0.24	1.06	0.070
	**Nulligravida (before current pregnancy)**	**0.24**	**0.07**	**0.81**	0.021*
Social distancing	**No formal education**	**0.20**	**0.07**	**0.61**	0.004*
	Unemployed husband	0.71	0.40	1.28	0.253
	Self-employed husband	0.74	0.37	1.46	0.381
	Low income (< 3 K SAR)	0.68	0.44	1.03	0.071
	**No. householders (1)**	**0.48**	**0.27**	**0.86**	0.013*
	**Second trimester pregnancy**	**1.54**	**1.10**	**2.15**	0.012*
	Medical comorbidity	0.71	0.45	1.13	0.149
Masking	**No formal education**	**0.32**	**0.10**	**0.99**	0.048*
	Unemployed husband	0.76	0.45	1.31	0.324
	**Self-employed husband**	**0.52**	**0.30**	**0.91**	0.022*
	**Low income (< 3 K SAR)**	**0.56**	**0.39**	**0.80**	0.002*
	No. householders (1)	0.66	0.35	1.26	0.206
	No. householders (3–4)	0.80	0.57	1.11	0.183
	Gravida 2–3	0.78	0.54	1.12	0.180
	Nulliparous	1.13	0.75	1.72	0.552
	Multiparous (4+)	1.31	0.78	2.19	0.309

## Discussion

### Summary of findings

Vulnerable populations constitute the weak link in public health actions. During the COVID-19 pandemic, protecting pregnant women from infection has the double benefit of preventing maternal and fetal complications. This nationwide study focused on the behavioral dimension of COVID-19 preventive strategy among pregnant women, and analyzed the determinants of adherence to the basic preventive measures, along with the associated socioeconomic, obstetrical and cognitive factors. Levels of self-reported compliance were remarkably high (≥ 90%), except for avoidance of contact with an infected person, to which almost 30% of the participants struggled adhering. Levels of adherence were significantly associated with levels of risk perception, notably the perceived severity of COVID-19 and perceived harmfulness for the baby. Analysis of socio-demographic factors highlighted the significance of education and economic status in determining adherence to preventive measures, which represents a potential inequity in the risk of COVID-19 infection.

### Compliance to preventive measures among pregnant women

Few studies in the literature explored compliance to preventive measures among pregnant women. Consistent with this study, a single-center study from India reported high levels of practice (92.7%) in preventive measures among 532 pregnant women visiting the outpatient obstetrics clinic in tertiary hospital. This was associated with relatively less levels of knowledge and attitude towards COVID-19 [23]. Similar results were observed in a multi-center Iranian study, which showed 97.3% of the 225 included pregnant women had adequate compliance to protective behaviors. However, it is to note that researchers only included women who can read and write which would skew the levels of compliance in favor of the educational level, explaining these high Fig. [24]. Conversely, a study from Nigeria showed relatively lower levels of adherence (78%) to preventive measures among 442 pregnant women, in addition to low levels of knowledge (48%) [25]. In Ghana, even lower compliance rates were reported for mask wearing (18%), hand washing (32%), and social distancing (22%) among 527 pregnant women visiting the antenatal care clinics in 16 centers. Authors found that knowledge about COVID-19 symptoms and transmission routes were strong predictors for adherence to most preventive measures including mask wearing, hand washing, and social distancing [26]. Two other studies from Ethiopia showed compliance rates as low as 45% and 56.1% among 678 and 396 pregnant women, respectively [27, 28]. Both studies found knowledge about COVID-19 to be a significant predictor for compliance. These differences between the studies are probably due to cultural factors as well as varying levels of awareness and enforcement of the preventive measures by the government. In Saudi Arabia, drastic measures have been implemented nationwide, at an early stage of the pandemic. These were supported with massive awareness campaigns notably to strengthen public trust and compliance in the preventive strategy. A national cross-sectional study conducted during the first months of the crisis showed that more than 95% of the participants agreed on majority of the preventive and restrictive measures. The same study showed high levels of compliance with different preventive measures notably hand washing/disinfection and social distancing [29]. Likewise, another national survey including 2393 participants from the general population showed that hand washing and social distancing were adequately adopted by 96% and 98% of the participants, respectively [30]. However, both the previous studies reported relatively lower levels of compliance to face masking (~ 56%). With the exception of face masking, these figures compare well with the subpopulation of pregnant women from the present study. This difference may be explained by differences in the study periods, resulting in different levels of public response to preventive measures. Another explanation may be that the two previous studies in the general population were carried out online, while the present study was conducted at the clinic, which may induce social desirability bias.

### Risk perception and its association with preventive behavior

The authors found that compliance to preventive measures was significantly associated with perceptions and beliefs about COVID-19 severity, infectiveness, and harmfulness. However, because 31% of the participants did not complete the risk perception questionnaire, the risk perception parameters were not included in the multivariate models of compliance. This high proportion of non-responders prevented from carrying out imputation methods for missing data. Additionally, further analysis showed significant socio-demographic differences between responders and non-responders to the risk perception questionnaire, notably in terms of region, economic and educational status, and obstetrical factors including gravida and semester. This analysis showed that non-responders were more likely to live among a higher number of householders (38.9% vs. 31.9%, p = 0.001) and have lower income (p = 0.015) and educational status (p < 0.001), higher gravida (p = 0.009) and parity (p = 0.001), and being in an earlier stage of pregnancy (15.9% vs. 12.0%; p = 0.029), compared with responders. However, non-responders were comparable to responders in terms of age (p = 0.125), nationality (p = 0.190), number of school children (p = 0.082), level of pregnancy risk (p = 0.709), and past medical history (p = 0.661). This comprises a high risk of selection bias, as such sociodemographic factors may significant determinants of risk perception. These two reasons constituted methodological and statistical limitations to the inclusion of risk perception data in the multivariate model.

Nevertheless, univariate analysis demonstrated that undermining COVID-19 severity as a disease was associated with less compliance to all four preventive measures. On the other hand, participants who believed COVID-19 is a serious disease significantly had higher compliance rates compared to their counterparts. Likewise, perceived risk of infection was associated with higher compliance to hand hygiene and social distancing. There is substantial evidence demonstrating the association between risk perception and compliance with preventive measures in COVID-19. Majority of such data emanate from studies using theoretical cognitive and behavioral models that enabled designing the best communication strategies to enhance behavioral commitment to the public health measures among population. One of the interesting approaches is the Health Belief Model, which provides a conceptual framework to predict voluntary compliance to preventive behavior based on the individual’s perception of a disease severity and own susceptibility/vulnerability to this disease, in addition to perceived benefits from, barriers to and cues to action regarding the relevant preventive measures [31]. The application of this model in COVID-19 showed that variables of the Health Belief Model significantly predicted the behavioral change in terms of compliance to preventive measures [32, 33].

More specific to the study population, assessing perceived harmfulness to the baby of COVID-19 was of particular interest, although it could be considered a derivative of perceived vulnerability. In the present study, perceived harmfulness to the baby was associated with higher compliance to avoidance of contact with infected persons and face mask wearing. Women who believed COVID-19 was harmless for the baby represented 15% of the sample. In an Indian study, approximately half of the pregnant women perceived the risk of COVID-19 fetal transmission to be high, while a minority feared premature delivery or intra-uterine growth restriction or death as a consequence of a COVID-19 infection during pregnancy. Authors concluded that pregnant women ex-pressed more concern about transmitting the infection to their families and children than to their unborn babies [34]. This issue of mother’s perception and anticipation of fetal health risk is complex, irrespective of the nature of risk or disease, as it involves several psycho-emotional, societal, and cultural dimensions, besides the cognitive factors related to knowledge and awareness about the risks based on scientific evidence [36, 37]. In all cases, the role of physicians and other health professionals in the obstetrical care is to educate patients regarding both the maternal and fetal risks, in addition to the general risks, and to promote the adherence to preventive guidelines. This is to be tailored ac-cording to information requirements, considering eventual misinformation or overexposure to negative messages, while screening for an underlying maternal psychological disorder that may impact prenatal attachment and result in negative compliance to prevention [38]. This highlights the need for functional perception of COVID-19 maternal and fetal risks that promotes self-efficacy among pregnant women to adhere with preventive measures [39].

### Social determinants of compliance to preventive measures

One of the most important observations from the present study is the impact of social determinants of health on the prevention behavior against COVID-19. More specifically, lower education and low economic status were predictors for poor compliance reducing the probability of adherence by 20-80% depending on the preventive measure. Additionally, residence in the Northern Province was also a predictor for lower compliance to avoidance of contact with an infected person. These observations highlight the inequity in prevention behaviors resulting from social disadvantage. Comparable observations have been reported in other studies exploring compliance to preventive measures among pregnant women. In Ghana, higher education was independently associated with better compliance to face mask wearing, hand washing, and social distancing [26]. Similarly, in Ethiopia, higher maternal education was associated with better adherence to preventive measures [27]. In Iran, higher education was associated with higher levels of knowledge about COVID-19, but showed no significance with risk perception and protective behaviors [24]. In India, low education was associated with in-adequate knowledge and attitudes towards COVID-19, while it showed no significant association in practicing prevention measures [23]. In Nigeria, lower education was associated with lower adherence to preventive measures [25]. This emphasizes the importance of investigating the specific social determinants for each subpopulation to enable informing the public health decision-making regarding the most appropriate strategies to tackle inequalities in terms of uptake of preventive measures and the subsequent health outcomes.

### Limitations

The present study is limited by the relative underrepresentation of some regions of the country, notably the North and South provinces, which may impact the generalizability of the findings. This under-representativeness is mainly due to the low participation rate, which is probably related to low demography in these regions. Another underrepresented subgroup is expatriates, and this may be due to pregnant expatriates being more often followed at the private sector. Another limitation that may hinder the generalizability of the findings is the high percentage of highly educated participants, which is probably due to a selection bias related to the data collection method. Furthermore, the non-response rate to risk perception questions hindered the multivariate analysis, notably due to non-random effect as the missing data was significantly associated with the participant’s region and educational level. Another limitation is that levels of risk perception and compliance to preventive measure were assessed using participant’s self-declaration with a dichotomous scale, which is subject to social desirability bias, especially given that the interview was conducted at a care center. However, the simplified design of the questionnaire was intended to enhance the participation rate and shorten the time of completion of the questionnaire, thereby avoiding overloading the clinics’ spaces with visitors amid the pandemic period.

## Conclusion

The study found that pregnant women were highly compliant with basic preventive measures against COVID-19 early in the pandemic. Compliance was influenced by their perception of the severity and potential harm of the virus to their baby. It highlights the need for healthcare providers in obstetrics clinics to educate patients and tailor their approach based on individual information needs to promote self-efficacy in understanding the risks of COVID-19 for both mother and fetus. The research also underscores the impact of social disadvantage on adherence to preventive measures among this vulnerable group, emphasizing the importance of addressing social determinants of health to reduce inequalities, in terms of prevention efficiency, and improve health outcomes.

## Data Availability

The datasets used in the present study are available from the corresponding author on reasonable request.
